# Treating Disorders of Consciousness With Apomorphine: Protocol for a Double-Blind Randomized Controlled Trial Using Multimodal Assessments

**DOI:** 10.3389/fneur.2019.00248

**Published:** 2019-03-19

**Authors:** Leandro R. D. Sanz, Nicolas Lejeune, Séverine Blandiaux, Estelle Bonin, Aurore Thibaut, Johan Stender, Neal M. Farber, Ross D. Zafonte, Nicholas D. Schiff, Steven Laureys, Olivia Gosseries

**Affiliations:** ^1^GIGA Consciousness, University of Liège, Liège, Belgium; ^2^Coma Science Group, University Hospital of Liège, Liège, Belgium; ^3^CHN William Lennox, Groupe Hospitalier Saint-Luc, Ottignies-Louvain-la-Neuve, Belgium; ^4^Institute of Neurosciences, UCLouvain, Brussels, Belgium; ^5^Department of Physical Medicine and Rehabilitation, Harvard Medical School, Boston, MA, United States; ^6^Spaulding Rehabilitation Hospital, Massachusetts General Hospital, Brigham and Women's Hospital, Boston, MA, United States; ^7^Department of Neuroscience, University of Copenhagen, Copenhagen, Denmark; ^8^NeuroHealing Pharmaceuticals Inc., Newton, MA, United States; ^9^Feil Family Brain and Mind Research Institute, Weill Cornell Medical College, New York, NY, United States

**Keywords:** disorders of consciousness, unresponsive wakefulness syndrome, minimally conscious state, apomorphine, dopamine, mesocircuit, clinical trial, protocol

## Abstract

**Background:** There are few available therapeutic options to promote recovery among patients with chronic disorders of consciousness (DOC). Among pharmacological treatments, apomorphine, a dopamine agonist, has exhibited promising behavioral effects and safety of use in small-sample pilot studies. The true efficacy of the drug and its neural mechanism are still unclear. Apomorphine may act through a modulation of the anterior forebrain mesocircuit, but neuroimaging and neurophysiological investigations to test this hypothesis are scarce. This clinical trial aims to (1) assess the treatment effect of subcutaneous apomorphine infusions in patients with DOC, (2) better identify the phenotype of responders to treatment, (3) evaluate tolerance and side effects in this population, and (4) examine the neural networks underlying its modulating action on consciousness.

**Methods/Design:** This study is a prospective double-blind randomized parallel placebo-controlled trial. Forty-eight patients diagnosed with DOC will be randomized to receive a 30-day regimen of either apomorphine hydrochloride or placebo subcutaneous infusions. Patients will be monitored at baseline 30 days before initiation of therapy, during treatment and for 30 days after treatment washout, using standardized behavioral scales (Coma Recovery Scale-Revised, Nociception Coma Scale-Revised), neurophysiological measures (electroencephalography, body temperature, actigraphy) and brain imaging (magnetic resonance imaging, positron emission tomography). Behavioral follow-up will be performed up to 2 years using structured phone interviews. Analyses will look for changes in behavioral status, circadian rhythmicity, brain metabolism, and functional connectivity at the individual level (comparing before and after treatment) and at the group level (comparing apomorphine and placebo arms, and comparing responder and non-responder groups).

**Discussion:** This study investigates the use of apomorphine for the recovery of consciousness in the first randomized placebo-controlled double-blind trial using multimodal assessments. The results will contribute to define the role of dopamine agonists for the treatment of these challenging conditions and identify the neural correlates to their action. Results will bring objective evidence to further assess the modulation of the anterior forebrain mesocircuit by pharmacological agents, which may open new therapeutic perspectives.

**Clinical Trial Registration:** EudraCT n°2018-003144-23; Clinicaltrials.gov n°NCT03623828 (https://clinicaltrials.gov/ct2/show/NCT03623828).

## Introduction

Following severe brain injury, patients who emerge from coma may develop disorders of consciousness (DOC) such as the unresponsive wakefulness syndrome (UWS) or the minimally conscious state (MCS). UWS patients only present reflex behaviors (1) whereas MCS patients demonstrate unequivocal but inconsistent evidence of awareness through a wide variety of behavioral responses that can be demonstrated at the bedside. However, they are not able to functionally communicate or use objects (2).

### Treating the Severely Brain-Injured

There are currently no international guidelines to treat patients with chronic DOC and available therapies have demonstrated limited efficacy so far. Moreover, most of the available information to date arises from uncontrolled studies. Several non-pharmacological and pharmacological treatments have, however, shown positive preliminary results. Transcranial direct current stimulation (tDCS) on the dorsolateral prefrontal cortex (i.e., a non-invasive neurostimulation technique) could induce short-term behavioral improvements in 50% of patients in a chronic MCS, without similar effects observed in patients with UWS (3). When applied repeatedly for 5 consecutive days, tDCS elicited effects up to a week after the end of stimulations in 56% of MCS patients (4). Recent results suggest the applicability of this technique in a home-based setting (5), but further research is needed to confirm long-term efficacy and tolerance (6). Moderate behavioral improvements have also been observed after deep brain stimulation on the central thalamus, but only a limited fraction of patients were eligible for this intervention which is associated with significant side effects related to invasive brain surgery (7–9). Among pharmacological treatments, zolpidem (a non-benzodiazepine hypnotic that potentiates GABA_A_ receptors) could paradoxically improve behavioral responsiveness in severely brain-injured patients but effects were short-lasting and reported in only a small minority of patients with DOC (10, 11). Because of the role of dopamine as a general stimulatory neurotransmitter (12) and in maintaining wakefulness in circadian rhythms (13), several dopaminergic agents have been used in an attempt to stimulate UWS and MCS patients (14, 15), namely levodopa, bromocriptine, amantadine, and apomorphine (16).

Levodopa is a dopaminergic agent commonly used for the treatment of Parkinson's disease. Previous studies have shown positive behavioral effects in UWS patients after its administration (17, 18). The effects observed within 10 days of treatment included the recovery of command-following and reciprocal interaction. Bromocriptine is another dopaminergic agonist acting on postsynaptic dopamine D2 receptors, also used primarily as anti-Parkinsonian therapy. It has been associated with a higher rate of patients recovering from a post-traumatic UWS in a retrospective study (19). However, in a 6-week double-blind, placebo-controlled, crossover study, bromocriptine did not improve attentional skills in 12 conscious patients with moderate to severe traumatic brain injury (20). Last, amantadine is used as an antiviral and as an anti-Parkinsonian agent, acting as a weak antagonist of the NMDA-type glutamate receptor as well as increasing dopamine release and blocking its reuptake. One placebo-controlled study has shown its efficacy to increase recovery rate in UWS and MCS patients suffering from traumatic brain injury, although no difference with the placebo group was observed 2 weeks after washout (21). Based on these results, amantadine is currently the only drug recommended in the recently published American practice guidelines for DOC for the treatment of UWS and MCS patients between 4 and 16 weeks following brain injury (22).

### Apomorphine—A Promising Agent to Improve Recovery

Two small-sample uncontrolled studies have shown that apomorphine, a nonselective dopamine agonist with a high affinity for D2 receptors, may dramatically improve the outcome of patients with UWS and MCS (23, 24). While other agents increase the availability of natural dopamine by modulating synaptic reuptake rates, apomorphine exerts a direct action on dopamine receptors, which entails the activation of its target neurons regardless of axonal damages (25, 26). Its fast action, high bioavailability and subcutaneous administration allow accurate and steady drug blood levels (27). It is marketed with an indication for advanced Parkinson disease refractory to treatment, and has a well-documented safety profile, causing mainly minor and manageable side effects such as local skin nodules, nausea, and hypotension (28–30). All eight patients with traumatic brain injury treated with apomorphine showed significant behavioral improvements within 1 to 62 days of treatment (24). These patients had been previously treated with other dopaminergic stimulants without response, whereas apomorphine elicited significant effects. However, the lack of a control group did not allow to fully disentangle the responses due to apomorphine treatment of these patients from possible spontaneous recovery. In addition, no study has investigated the neural mechanisms underlying the action of apomorphine in patients with DOC, although some hypotheses have been formulated.

### The Mesocircuit Model—A Framework for the Role of Dopamine

The mesocircuit model (31) could help us understand the behavioral response to dopaminergic agents in severely brain-injured patients. This model explains the vulnerability of the anterior forebrain following multi-focal brain injuries that produce widespread deafferentation or neuronal cell loss. Decreased projections from the central thalamus and the frontal cortex cause an insufficient afferent drive on the dopaminergic medium spiny neurons of the striatum, which fail to actively inhibit the globus pallidus interna. The globus pallidus interna inhibits central thalamic neurons which in turn fail to sustain their activating projections on cortical areas (32). Impairment of these loops mainly relying on dopamine could lead to a broad synaptic activity decrease in areas responsible for the generation of consciousness (33). Abnormalities of dopamine neurotransmission, such as reductions in dopamine transporter and expression of D2 receptor, have been reported in patients after closed-head trauma, which supports the postulated central role of dopamine in severe brain injury (34–36).

**Figure 1:**
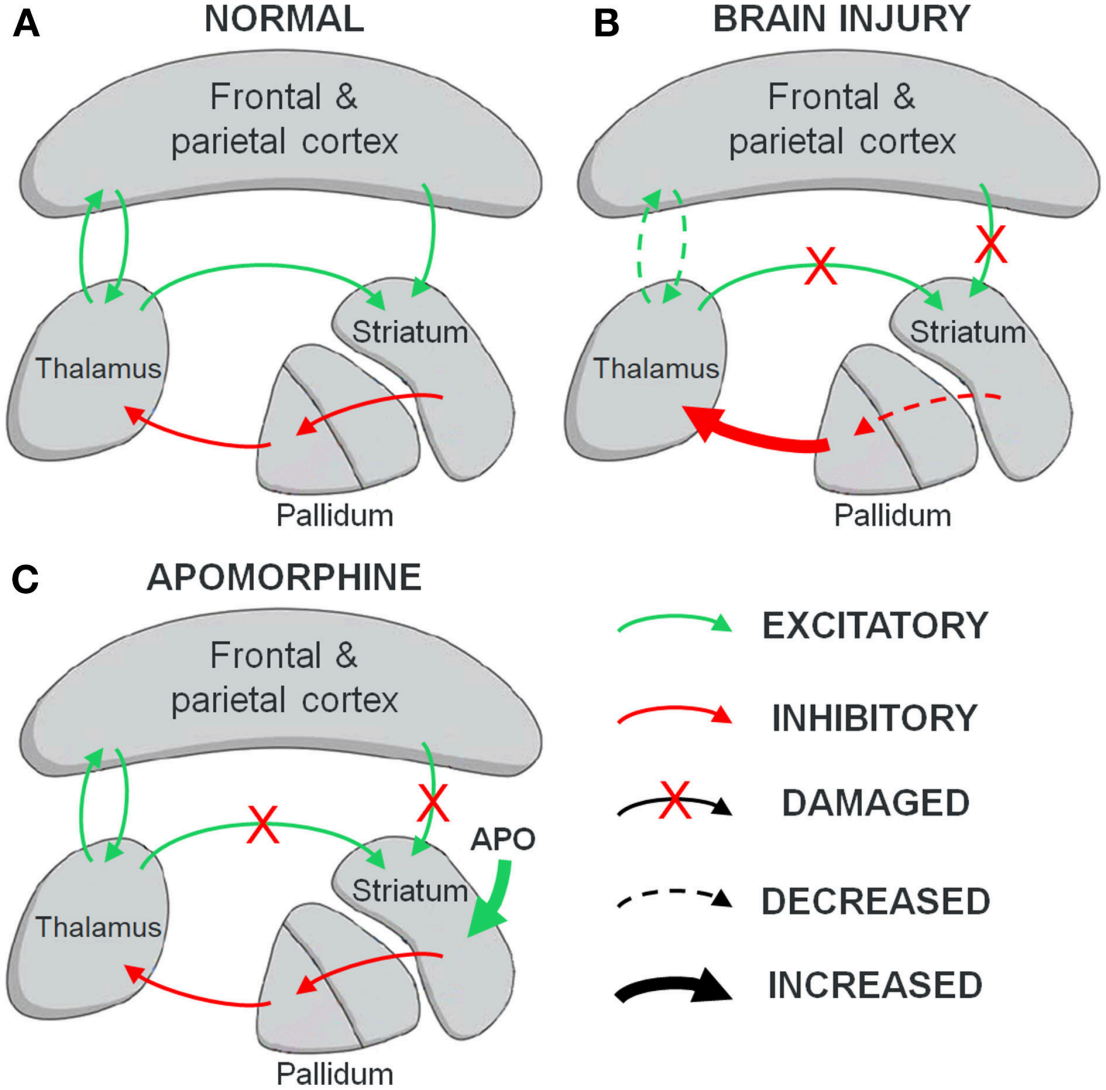
The mesocircuit hypothesis. **(A)** Normal wakeful condition. Dopamine neurons in the striatum inhibit the pallidum, which prevents it from inhibiting the thalamus. Thalamic projections activate cortical networks and get positive feedback in return. Excitatory inputs from both the cortex and the thalamus activate the striatum to maintain the loop. **(B)** Brain injury. Withdrawal of thalamostriatal and corticostriatal projections following widespread neuronal deafferentation leads to reduced activity of the striatum, resulting in an inhibition of thalamic activity and decreased cortical activation. **(C)** Postulated action of apomorphine (APO) on brain injury. The facilitating action of apomorphine on striatal dopamine neurons could substitute for the missing inputs and restore the inhibitory striatopallidal projections, thus freeing the thalamus and its output toward the cortex.

Treatment by exogenous dopaminergic agonists would hypothetically increase the firing rate of medium spiny neurons in the striatum and facilitate projections inhibiting the globus pallidus interna, restoring the loops between the cortex, the striatum, the globus pallidus interna and central thalamic nuclei [Figure 1; (37)]. In particular, the high density of D2-type inhibitory medium spiny neurons in the striatum suggests that agents with a higher affinity for this receptor, such as apomorphine, may be suitable therapeutic candidates (34). The postulated resulting central thalamic modulation would be in line with the previously described restoration of thalamo-cortical connectivity following spontaneous recovery from UWS (38), as well as clinical improvements obtained with deep brain stimulation of the central thalamus in MCS patients (9).

In the light of the above, we therefore propose to investigate the effects of apomorphine in patients with DOC in a multimodal placebo-controlled clinical trial including neuroimaging and neurophysiology techniques.

### Study Objectives

This research aims to (1) evaluate the effect of apomorphine treatment for the recovery of consciousness among patients with UWS and MCS in a placebo-controlled setting to better characterize the nature, size and duration of this effect, (2) better define the phenotype of potential good candidates to apomorphine treatment and identify a set of biomarkers that correlate with responsiveness (or non-responsiveness) to the therapy, (3) evaluate the tolerance and the occurrence of side effects of daily subcutaneous apomorphine infusions in this vulnerable population, and (4) investigate the neural mechanisms underlying the action of apomorphine treatment in severe brain injury using neurophysiological and neuroimaging assessments to detect brain activity changes, with the mesocircuit model as theoretical framework. Not only will these results help to define clearer guidelines in the treatment of patients with DOC, but they will also allow a better understanding of how dopamine networks are involved in consciousness and its impairments.

### Study Hypotheses

Our main hypothesis is that apomorphine treatment will increase responsiveness and induce behavioral changes in a significant fraction of the patient population. We expect that these changes will in some cases improve the patients' diagnosis, according to the Coma Recovery Scale-Revised (CRS-R) guidelines (39).

We surmise that the recovery of physiological circadian sleep-wake cycles, measured by actigraphy, core body temperature monitoring and sleep architecture assessed by night electroencephalography (EEG), may be correlated with behavioral responsiveness to the therapy. Total movements as measured by actigraphy may also increase in responding patients.

Based on the mesocircuit model, we hypothesize that responders to the treatment will show an increase of axonal projections from the striatum to the globus pallidus interna, restoring central thalamic activity, and resulting in higher integration of neuronal activity across the cortex. We postulate that these changes will translate into modifications of brain metabolism and functional connectivity. We expect an improvement in brain metabolism measured by fludeoxyglucose positron emission tomography (FDG-PET), with significant increases of whole brain glucose standardized uptake value after treatment, in particular in the striatum, thalamus and frontoparietal areas. We also predict a modulation of functional connectivity by apomorphine treatment measured by resting-state functional magnetic resonance imaging (fMRI) seed-based analyses as well as increased resting-state EEG dynamic connectivity and spectral power metrics (e.g., mean alpha spectral connectivity, participation coefficient variance, delta modularity).

## Methods

### Study Design

This is a prospective double-blind randomized parallel placebo-controlled trial. The trial will be preceded by an open-label pilot phase on six patients with the same study design except for the absence of a control group, to assess feasibility.

### Population and Recruitment

We will include 48 patients with DOC following severe acquired brain injury. All participants will be inpatients undergoing neurological rehabilitation in the post-coma unit. Patients will be assessed by the referring neurologist at the time of their admission and eligibility for the study will be determined. An accredited examiner will determine the diagnosis using standardized CRS-R criteria.

The inclusion and exclusion criteria are as follows:

Inclusion criteria:

– 18–55 years old.
– Clinically stable, independent of medical ventilators for respiration.
– Diagnosed as in an UWS or MCS according to the international criteria and based on at least 2 consistent CRS-R in the last 14 days (one CRS-R in the last 7 days).
– More than 4 weeks from onset of the injury.
– Informed consent from legal representative of the patient (and from the patients in the event they recover legal capacity).

Exclusion criteria:

– More than 6 months from onset of the injury.
– Use of dopamine agonists or antagonists (e.g., amantadine, bromocriptine, levodopa, pramipexole, ropinirole, amphetamine, bupropion, methylphenidate/risperidone, haloperidol, chlorpromazine, flupentixol, clozapine, olanzapine, quetiapine) during the last 2 weeks or 4 half-lives of the drug.
– Use of neurological medications other than anticonvulsant or anti-spasticity drugs during the last 2 weeks or 4 half-lives of the drug.
– Use of drugs with known significant prolongation of the QT interval (e.g., class 1 antiarrythmics, sotalol, macrolides, quinolones, antipsychotic drugs, tricyclic antidepressants, methadone, chloroquine, quinine) during the last 2 weeks or 4 half-lives of the drug.
– A corrected QT interval over 480 ms (calculated using Bazett's formula on a standard 12-lead ECG, recorded in the last 14 days) or other risk factors for arrhythmia (congestive cardiac failure, severe hepatic impairment, or significant electrolyte disturbance).
– A history of previous neurological chronic disorder other than related to their acquired brain injury.
– Contraindication to MRI, EEG, or PET (e.g., electronic implanted devices, external ventricular drain).
– Use of nitrates or other vasodilators, central nervous system acting agents such as barbiturates, morphine, and related drugs (relative exclusion criterion).

### Procedure

**Figure 2:**
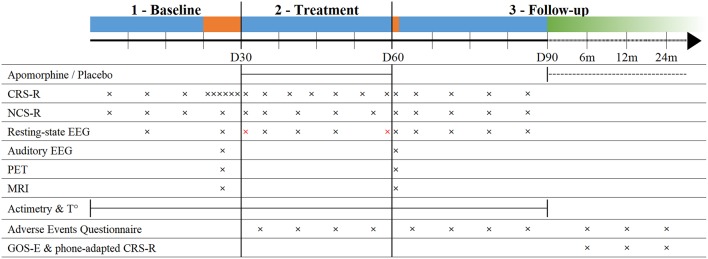
Timeline of the study protocol. Blue segments: inpatient phases; Orange segments: multimodal assessments; Green segment: outpatient remote follow-up; dashed line: optional treatment extension; CRS-R, Coma Recovery Scale-Revised; NCS-R, Nociception Coma Scale-Revised; EEG, electroencephalography; PET, positron emission tomography; MRI, magnetic resonance imaging; T°, body core temperature; GOS-E, Glasgow Outcome Scale-Extended; Red crosses, 24 h EEG.

Twenty-four of the included patients will be randomly assigned to the apomorphine group and twenty-four to the placebo group. Random allocation will be performed using a computerized random number generator to achieve blocked randomization. The assignment sequence will be generated and known only by two main investigators not involved in patient assessment, who will communicate the assigned intervention to the referent site pharmacist for each patient after enrollment. The pharmacist will then prepare the product and deliver it to the care providers in a neutral packaging. The assignment to interventions as well as the size of the blocks will be concealed to the patient, their relatives, the care providers, and all investigators performing patient recruitment, bedside assessments and data analysis. Concealment will be maintained after assignment during baseline assessment, treatment, and inpatient follow-up, following a double-blind design. Investigators performing data analyses will remain blind for the whole duration of the study. Figure 2 illustrates the clinical protocol procedure.

#### Baseline Assessment

The initial 3 weeks (day 1 to day 21) after inclusion in the study (“baseline”), patients will be assessed once a week using the CRS-R and the Nociception Coma Scale-Revised [NCS-R, (40)] in order to have a reliable diagnosis and a measure of pain levels. One week before starting the treatment (day 22 to day 28), patients will undergo a multimodal assessment with CRS-R, NCS-R, MRI, FDG-PET and high density EEG during resting state and during auditory paradigms. Actigraphy and body temperature data will be recorded continuously from day 1 up to day 90.

#### Treatment Phase

From day 31 to day 60, patients will be administrated apomorphine or placebo. Based on the dosing schedule used in previous studies (24), apomorphine will be administered subcutaneously 12 h per day during daytime (~09:00 am−09:00 pm) with an initial increase of doses: 1 mg/hour on day 31, 1.5 mg/hour on day 32, 2 mg/hour on days 33 and 34, up to 4 mg/hour from day 35 up to day 42 if no substantial adverse effect is noted. On day 43, depending on the patient's tolerance, clinical response and expected benefit, the referent physician will be given the choice to increase the infusion rate to 6 mg/hour or to keep it at 4 mg/hour until day 60. If the referent physician considers necessary at any time during the treatment (e.g., in case of significant side effects), the infusion will be decreased to the prior rate or stopped. A subsequent increase of dose will only be considered after clinical stability for a minimum of 7 days. Placebo will consist of isotonic saline infusions delivered via subcutaneous apomorphine pump. Placebo and apomorphine will be conditioned with similar packaging, labeling and product aspect.

Two days prior to the initiation of treatment, 20 mg t.i.d of domperidone will be administered to all patients to reduce possible side effects such as nausea and vomiting. Antiemetics will be maintained for a minimum of 7 days before tapering off. It can also be continued beyond this point at equal or lower dosage if the referent physician deems necessary. With these precautions, side effects (nausea, vomiting, and hypotension) related to apomorphine administration will be significantly reduced (28). A standard 12-lead ECG will be performed before the initiation of treatment to rule out long QT interval, as both apomorphine and domperidone may prolong QT interval and increase the risk for arrhythmias (41, 42).

During treatment phase, behavioral assessments (CRS-R and NCS-R) and resting-state EEG will be performed weekly. Sleep EEG will be recorded once just before treatment initiation and once after treatment termination.

#### Follow-Up

Immediately after the cessation of the treatment (day 61 or earlier if treatment terminated), all patients will undergo another multimodal assessment (see Baseline Assessment). Between day 61 and day 90, patients will be assessed weekly with the CRS-R, NCS-R, and resting-state EEG (Figure 2).

The presence of adverse effects will be recorded weekly from the initiation of treatment (day 31) until the end of the washout follow-up (day 90) using an Adverse Events Questionnaire form (Supplementary Data 1).

If, after day 90, the patient's clinical state deteriorates after treatment withdrawal to the point of a negative CRS-R diagnosis change, the referent physician will be given the option to resume apomorphine treatment using the last prescribed dose after progressive escalation. The duration of the extension will be determined by the referent physician based on clinical judgment.

Remote follow-up evaluations by structured phone interviews will also be carried out at 6 months, 1 and 2 years post-evaluation using the Glasgow Outcome Scale-Extended (43), a phone-adapted version of the CRS-R and the Adverse Events Questionnaire.

### Instruments

We will employ the following clinical, neurophysiological, and neuroimaging techniques:

#### Apomorphine or Placebo Treatment

Patients will be randomized at inclusion to receive either apomorphine hydrochloride at a 5 mg/mL concentration, as available in pre-filled syringes, or normal saline placebo (sodium chloride 9 mg/mL) matched for fluid volume and aspect. Apomorphine will be administered by subcutaneous infusions using a Crono APO-go III pump (Britannia Pharmaceuticals, Reading, UK). The tank of the pump will be filled by the study pharmacist with either apomorphine or placebo according to the patient's assigned intervention, and a clinical trial label will be displayed on the pump. Subcutaneous infusions lines will be placed using a 20- to 30-gauge metal butterfly needle and the infusion site will be chosen at the discretion of the clinical staff, according to the manufacturer's guidelines (e.g., below umbilicus, upper outer thighs, shoulder). The infusion site will be changed at the beginning of each day using a new sterile needle, or more often if local adverse effects are noted during the infusion.

The treatment will start with a titration phase designed to determine the optimal tolerated dose for each patient. The maintenance phase will then follow, for a total of 30 days of treatment. Infusion will take place 12 h a day during daytime (~09:00 am−09:00 pm) to avoid perturbing sleep-wake cycles.

#### Behavioral Assessments

The CRS-R is the gold standard behavioral scale for diagnosis of consciousness (44). This scale comprises 23 items, separated into six subscales, assessing the visual, motor, auditory, and oro-motor/verbal functions as well as communication and arousal. The diagnosis relies on the presence or absence of specific items for each diagnostic entity, rather than the total calculated score (39). A modified score has been developed to detect conscious awareness with a higher sensitivity and specificity than the total score, using a single metric (45).We will use the validated French version of the CRS-R for French-speaking patients (46).

The NCS-R was created in order to detect pain in DOC patients. This scale assesses reactions to noxious stimulation and scores for motor, verbal, and facial responses. Its total score ranges from 0 (no pain) to 9 (maximal pain) (40).

The Glasgow Outcome Scale-Extended is used to classify global outcomes in traumatic brain injury survivors within 8 categories: dead, vegetative state, lower severe disability, upper severe disability, lower moderate disability, upper moderate disability, lower good recovery and upper good recovery (43). A structured phone interview version of the scale will be used to allow remote follow-up of recovery.

The phone-adapted version of the CRS-R is a tool developed to assess the most significant items of the CRS-R (i.e., key criteria for the diagnosis of MCS and emergence of MCS) through a structured phone interview based on the observations of relatives and caregivers during the last 2 weeks and provides a diagnosis.

The Adverse Events Questionnaire (Supplementary Data 1) is designed to probe the most frequent adverse events associated with the administration of apomorphine and allows the referent physician to report the nature, the severity and the probable causality of new adverse events. It was created for this trial using available information on the most frequent adverse events associated with apomorphine (28, 42).

#### Circadian Rhythm

Circadian rhythmicity describes the cyclic variations of body parameters over a 24 h period. This process is regulated by light intensity changes which triggers melatonin release during the night and its inhibition during the day. Among patients with DOC, it is still unclear how the circadian rhythm is altered and more importantly how it affects their sleep and cognition (47, 48). Variations in spontaneous movements, body core temperature, and EEG activity can be used in order to monitor biomarkers reflecting the circadian rhythm throughout day and night. From the beginning of baseline assessment (day 0) to the end of washout follow-up (day 90), a wrist actimeter (MotionWatch 8, CamNtech Ltd., Cambridge, UK) will measure the patient's spontaneous movements continuously and non-invasive temperature sensors (iButtons, Maxim Integrated, San Jose, CA, USA) will record core body temperature at 4 points of the body (subclavicular and interior malleola), as well as room temperature (49). Twenty-four-hour EEG recordings (23 channels, Grass Comet Plus, Natus Medical Inc, Pleasanton, CA, USA) will be performed before and after apomorphine treatment, in order to analyze sleep architecture changes (50).

#### EEG

EEG is the recording of electrical brain activity along the scalp. EEG measures voltage fluctuations resulting from ionic current flows within ensembles of neurons. In clinical context, event-related potentials (ERPs) involve averaging the EEG activity time-locked to the presentation of a stimulus. The presence of specific auditory ERPs such as the mismatch negativity of the P300 is well-known to reflect the ability to cognitively process sounds (51). The recording of these effects has been used as a noninvasive tool to assess levels of consciousness and predict outcomes of DOC patients (52). EEG activity will be recorded during resting-state and during a “local-global” oddball auditory paradigm, which allows an orthogonal manipulation of automatic and conscious responses to irregularity (53). Multivariate classifiers using machine learning were recently developed to stratify patients with DOC using an array of individual EEG metrics reflecting ERPs, spectral measures, information, and connectivity (54, 55). In addition, the resting-state data can be used to evaluate spectral connectivity using graph-theoretic metrics, which have proven to correlate with behavioral recovery of patients (56).

We will use a high-density EEG device (256 channels, Electrical Geodesics Inc, Eugene, OR, USA) and a conventional EEG device (23 channels, Grass Comet Plus, Natus Medical Inc, Pleasanton, CA, USA).

#### FDG-PET

FDG-PET is a technique where radioactively labeled isotopes are incorporated in certain chemical substances (tracers) and infused in the blood. By simultaneous external detection of the radioactively-emitted particles, the uptake of the tracer can be calculated across the brain. With the FDG tracer, glucose transport and metabolism can be measured. The patient will lie still in a darkened room for 15 min before tracer injection. After the injection, high-density EEG recordings will be performed during the cerebral uptake of the tracer. PET scanning will be performed 30 min after the tracer injection. If the patient is agitated before the scanning, sedation will be initiated using the lowest possible doses in order to reduce head motion and obtain better-quality images (PET images are sensitive to head motion). The sedation will be administered after the tracer uptake period such that measured brain metabolism is not affected. Sedation will last only the time of the exam and an anesthesiologist will be present during the whole exam. FDG-PET scanning will be performed using a Gemini TF PET/CT scanner (Koninklijke Philips N.V., Amsterdam, the Netherlands). PET acquisition procedures and image processing will be similar to previously described methods (57, 58).

#### Magnetic Resonance Imaging (MRI)

Structural MRI enables visualization of internal structures of the brain. Structural sequences (T1, T2-FLAIR and diffusion tensor imaging sequences) provide information on the anatomy of the brain and a voxel-based morphometry approach will allow to measure the loss of gray matter volume compared to healthy controls (59). Functional MRI will also be acquired with sequences that can evaluate the resting-state activity and the functional connectivity of different brain networks (e.g., default mode, auditory, visual, salience, motor networks) (60, 61). MRI images are sensitive to head motion, therefore if the patient is restless before the scanning, sedation will be administered by an anesthesiologist. MRI will be performed with a 3T MAGNETOM Vida MRI scanner (Siemens, Munich, Germany).

### Electronic Data Collection and Management

All information collected during this study will be kept confidential. The data will be pseudo-anonymized and listed under an ID-code only accessible to researchers at the GIGA Consciousness, and protected by a firewall. Participants will have a right to inspect, correct and request the deletion of their personal data at any time for 20 years after the inclusion in the trial. Data management will comply with the General Data Protection Regulation (EU 2016/679) and a specific information sheet will inform patients or their legal representative of the nature of collected data and their rights regarding these data.

### Statistical Analyses

Analyses will focus on the detection of changes induced by apomorphine treatment at the individual level (comparing data before and after treatment) and at the group level (comparing the placebo arm to the treatment arm). Along with the nature of the assigned intervention, age, etiology, time since injury and diagnosis will be included as additional independent variables in the regression analyses as they have proved to influence the prognosis of patients with DOC (62). CRS-R diagnosis will be used as primary outcome measure. Secondary outcomes will include metrics on different levels: (1) behavioral assessments (CRS-R total score and modified score, NCS-R, GOS-E, phone-adapted CRS-R), (2) brain metabolism (PET scan), (3) functional connectivity (resting-state fMRI, resting-state high-density EEG), (4) circadian rhythm (actigraphy, core body temperature, sleep architecture), and (5) drug safety (Adverse Effects Questionnaire).

Statistical analysis of PET and fMRI data will be performed with statistical parametric mapping (SPM12, https://www.fil.ion.ucl.ac.uk/spm/) and CONN functional connectivity toolbox (http://www.nitrc.org/projects/conn/) (60, 63). Quantification of PET signal will use methods based on standardized uptake values of FDG (64). MRI functional connectivity will be probed with the seed-voxel approach, which determines temporal correlations and anti-correlations between regions of interest and the time course from all other brain voxels (65, 66). EEG data analysis will use EEGLAB (https://sccn.ucsd.edu/eeglab/) and FieldTrip (http://www.fieldtriptoolbox.org/) for the estimation of spectral power within fixed bands, and dynamic connectivity will be estimated using median spectral connectivity and graph-theoretic topology metrics such as clustering coefficient, path length, modularity and participation coefficient (56). “Local-global” EEG recordings will be analyzed with a multivariate classifier based on machine learning using an array of individual EEG metrics (54). Results will be corrected for multiple comparisons and considered statistically significant when *p* < 0.05. Significant results will be examined in terms of relevance to the treatment administered to determine whether they carry clinical importance in the context of this clinical trial.

### Power Study

The sample size for this clinical trial was determined using a study power calculation (G^*^Power, UCLA, CA, USA) for a two-sampled *t*-test based on the best available data regarding the effect size of apomorphine treatment. In the largest study to date for this indication (24), 75% of patients (6/8) reached a status of moderate disability or good recovery one year after treatment, while 24% of subjects attained this status at the same time point in a roughly comparable cohort of 443 patients with DOC following traumatic brain injury (67). To account for the dissimilarities between these two populations and the possible overestimation of the treatment effect in Fridman's study due to small sample size, a 1% type-1 and 20% type-2 error rate threshold was set, resulting in an ideal sample size of 42 subjects. Assuming a drop-out rate of 12.5%, in line with previous studies conducted in our department, we set the sample size to 48 patients.

### Dissemination of Results

All collected data will be kept anonymously in a protected database at the University of Liège, Belgium, in compliance with the (EU) 2016/679 General Data Protection Regulation. Anticipated dissemination of results includes scientific publications in international peer-reviewed journals, with open access on institutional repositories as required by Belgian legislation. The publication plan forecasts one first article after the initial open-label pilot study. After completion of the inpatient phase of the trial, we aim to deliver one article reporting behavioral (CRS-R, NCS-R), brain metabolism (FDG-PET), and drug safety results, one article reporting brain connectivity (MRI and EEG) and one article on circadian rhythm (core body temperature and actigraphy) data. An additional article reporting the long-term outcomes of participants after full completion of the trial is also expected.

## Discussion

Patients with chronic DOC are too often neglected by healthcare infrastructures and health insurance systems, which leads to a frequent suboptimal medical management of their condition (68). This increases the risk of both misdiagnosis (69), and failure to offer adequate and targeted treatment. Indeed, it has been demonstrated that the few available pharmacological therapies have limited efficacy to certain specific patient sub-populations (70). For these reasons, it is crucial that further research verifies the effect of available treatments within a controlled setting, and defines more clearly the characteristics of potential therapy responders. This protocol describes the first randomized clinical trial on the efficacy of apomorphine for the recovery of consciousness using multimodal assessments. It aims to confirm preliminary results observed in small-sample, non-controlled studies, and to better identify the functional phenotype of responders. This study will use the anterior forebrain mesocircuit model (31) as a framework to understand the action of dopaminergic agents for the restoration of consciousness. We will use neuroimaging to test the hypothesis that apomorphine modulates these striato-pallido-thalamo-cortical loops through a dopamine-dependent action (23). As such, this trial will bring direct evidence to challenge the mesocircuit model and shed new light on the neural mechanism of dopaminergic treatment for chronic DOC. Pinpointing neuroimaging changes induced by apomorphine treatment will allow us to compare our results with available evidence on the action of other pharmacological therapies for DOC. Indeed, it has been hypothesized that “awakening” drugs acting on diverging neurotransmitter systems (dopamine, GABA) may in fact operate through a common activating pattern restoring the mesocircuit functional loops (71). This model would be supported by the identification of neuroimaging features in responders to apomorphine treatment similar to those described for zolpidem and amantadine (16).

The main potential pitfall of this clinical trial is a possible overestimation of the treatment effect size and response rate based on available literature, due to the non-controlled nature of previous studies. This would lead to an inaccurate estimation of the necessary sample size, which may lead to insufficient power. An extension of the population size is planned if intermediate analyses reveal an effect size substantially lower than expected. Unexpected severe adverse effects are very unlikely given the well-documented safety profile of apomorphine and the extensive experience of its use in Parkinson's disease. Safety of the participants will be maximized by the use of the Adverse Events Questionnaire, the requirement to report immediately any severe adverse event and the possibility to adapt apomorphine and domperidone dosage in case of low tolerance.

Our findings may open new paths in the development of treatments targeting specific brain networks or receptor subtypes, and could reshape the therapeutic landscape for this challenging patient population in urgent need for better healthcare.

## Ethics Statement

Written informed consent will be systematically obtained from patients or their legal representative. They will be adequately informed on objectives, methods, and potential risks of the study. The written document of information and consent will include the name and contact information of the investigators in charge of the study, including their phone numbers. The information form will also contain a paragraph indicating that the investigators in charge of the study have insurance that will, under Belgian law of 2004, cover accidental damages. The legal guardian will be informed that they can choose not to participate in the study, and may at any time and without given cause withdraw from the study. Should the subject recover sufficient capacity for discernment during the trial, they will be informed that the study has been or is being performed, and consent will be obtained from the patient. This study was approved by the Hospital and University of Liège Ethics Committee and the William Lennox Neurological Hospital Ethics Committee (Belgian identifier B707201731937), as well as the Belgian Federal Agency for Medicines and Health Products (EudraCT identifier 2018-003144-23).

## Author Contributions

LS participated in conception and design of the study, coordination of trial, ethical committee procedures, clinical trial registration, and manuscript writing. NL participated in study design and local management of the study in neurological rehabilitation centers. SB and EB participated in protocol design and will participate in patient assessments. NF provided expertise regarding the clinical trial material and assisted clinical trial registration procedures. AT, JS, RZ, NS, and SL participated to study conception, design, and defined its theoretical framework. OG was involved in study conception, design, oversight of study implementation, ethical committee and registration procedures, and helped in manuscript writing. All authors contributed to manuscript revision.

### Conflict of Interest Statement

NF is an officer and board member of NeuroHealing. The remaining authors declare that the research was conducted in the absence of any commercial or financial relationships that could be construed as a potential conflict of interest.
