# Supplementary material for: Treating Disorders of Consciousness With Apomorphine: Protocol for a Double-Blind Randomized Controlled Trial Using Multimodal Assessments
Source: Front Neurol. 2019 Mar 19;10:248. doi: 10.3389/fneur.2019.00248 (PMC6433751; doi:10.3389/fneur.2019.00248)
Supplement: Supplementary file 1 [file Table_1.docx]

Supplementary Material

# Supplementary Data 1: Adverse Events Questionnaire

| Has the patient presented, since last AE questionnaire, |  |
| --- | --- |
| -Nausea or vomiting? | 🞎 Yes* 🞎 No |
| -Sleepiness or sedation? | 🞎 Yes* 🞎 No |
| -Induration or subcutaneous skin nodules at injection site? | 🞎 Yes* 🞎 No |
| -Other adverse events? | 🞎 Yes* 🞎 No  If yes: …………………………………... |
| -Any severe adverse event? | 🞎 Yes** 🞎 No  If yes: …………………………………... |
| -Any change of medication (excluding apomorphine and domperidone normal schedule)? | 🞎 Yes*** 🞎 No  If yes, indicate which treatment is concerned:…………………………………... |

* If “Yes”, fill in the specific form for the adverse events

** If “Yes”, fill in the specific form for the severe adverse events

*** If “Yes”, update the form “Current Treatment”
